# A first WHO reference reagent for the detection of anti‐human platelet antigen‐15b

**DOI:** 10.1111/vox.13167

**Published:** 2021-06-23

**Authors:** Giles Sharp, Anthony Poles, Lucy Studholme

**Affiliations:** ^1^ Biotherapeutics Division National Institute for Biological Standards and Control (NIBSC) Potters Bar UK; ^2^ Histocompatibility and Immunogenetics NHSBT Filton UK

**Keywords:** CD109, HPA‐15b, MAIPA, platelet alloantibody detection, reference reagent

## Abstract

**Background and Objectives:**

Alloantibodies to human platelet antigen‐15b (anti‐HPA‐15b) have been detected in mothers with foetal–neonatal alloimmune thrombocytopenia and in multiply transfused patients. Assays used to detect this antibody, which aids in disease diagnosis, can be unreliable and vary in sensitivity. The objective was to generate a stable, lyophilized anti‐HPA‐15b preparation and evaluate its suitability as a World Health Organization (WHO) reference reagent for use in the quality control of platelet alloantibody detection assays. Results from an international collaborative study to evaluate the preparation were used to assign a minimum potency at which laboratories can be expected to detect the antibody.

**Materials and Methods:**

Recalcified plasma containing anti‐HPA‐15b was aliquotted, lyophilized and coded 18/220. Twenty‐five laboratories in 16 countries tested doubling dilutions of the reconstituted material in glycoprotein‐specific assays such as the monoclonal antibody–specific immobilization of platelet antigen assay and reported the last positive (or endpoint) dilution.

**Results:**

Twenty‐four laboratories (96%) detected antibodies with HPA‐15b specificity in preparation 18/220. Reported endpoint dilutions were normally distributed with a modal dilution of 1 in 16 and ranged from 1 in 2 to 1 in 128. Only two laboratories (8%) failed to detect anti‐HPA‐15b at 1 in 8 dilution.

**Conclusions:**

When diluted 1 in 8, most laboratories detected anti‐HPA‐15b in preparation 18/220 using HPA‐15bb platelets but not with HPA‐15aa platelets. The participants agreed this to be an appropriate dilution for assignment as the minimum potency. In October 2020, the WHO Expert Committee on Biological Standardization approved 18/220 as an International Reference Reagent.

## INTRODUCTION

To date, a total of 41 platelet alloantigens have been defined serologically, of which 12 are grouped in six bi‐allelic systems (human platelet antigens; HPA ‐1, ‐2, ‐3, ‐4, ‐5, ‐15) [1, 2]. The molecular basis of the 41 antigens has been resolved, and in all but one (HPA‐14b), the difference between self and non‐self is defined by a single nucleotide polymorphism in the gene encoding the relevant membrane glycoprotein [2]. Alloantibodies against HPAs are involved in foetal–neonatal alloimmune thrombocytopenia (FNAIT), a disease in which foetal/neonatal platelets are destroyed by IgG alloantibodies from the incompatible, HPA‐sensitized mother. HPA antibodies are also involved in platelet transfusion refractoriness (PTR), the repeated failure to achieve the desired level of blood platelets following transfusion and in post‐transfusion purpura (PTP), the delayed reaction to a transfusion caused by recipient antibodies to platelet antigens. Identification of the HPA antibody specificity is essential to the diagnosis and treatment of the patient. In severe cases of thrombocytopenia requiring treatment with a platelet transfusion, it is important that the transfused platelets are negative for the target alloantibody specificity [3, 4, 5, 6].

A variety of techniques are in use across the world to detect HPA antibodies. The monoclonal antibody–specific immobilization of platelet antigen (MAIPA) assay is considered the gold standard method for the determination of platelet (IgG) alloantibody specificity. The method involves using specific HPA‐typed platelets to capture alloantibodies in human serum/plasma. Mouse monoclonal antibodies specific for glycoproteins on which the platelet antigens of interest are located are used to capture the platelet antigen—alloantibody complexes after solubilization of the platelets. This design ensures the accurate identification of alloantibody specificity and avoids false‐positives resulting from, for example, HLA antibodies. The MAIPA does not distinguish between IgG alloantibody subclasses in patient samples. To date, there are three World Health Organization (WHO) reference reagents established (anti‐HPA‐5b, anti‐HPA‐3a and anti‐HPA‐1a) [7, 8, 9], which are used for assay quality control. These reagents are used to validate the minimum sensitivity of tests for the respective HPA antibodies.

In addition to HPA‐1a, ‐3a and ‐5b, HPA‐15 is of clinical relevance. Studies have shown this antigen to be as immunogenic as HPA‐5, and alloantibodies against HPA‐15b can be detected in patients receiving multiple transfusions and mothers with FNAIT [10, 11, 12]. The platelet‐based methods used for the detection of anti‐HPA‐15 antibodies can be unreliable and vary in their sensitivity because CD109, the glycoprotein on which HPA‐15b is located, is expressed in low numbers by platelets and is labile [13, 14]. An anti‐HPA‐15b minimum potency reference reagent would allow clinical laboratories to validate their methods. The need for this important reference material was emphasized in the proficiency testing scheme organized by National Institute for Biological Standards and Control (NIBSC) in 2017 showing that anti‐HPA‐15b detection in the samples distributed was generally poor. Furthermore, it was concluded in a report by the Platelet Immunobiology Working Party of the International Society of Blood Transfusion (ISBT) in 2018 [15] that a more standardized approach to the CD109 MAIPA is required. The purpose of this study was to produce and evaluate a candidate WHO anti‐HPA‐15b reference reagent (minimum potency) for use as a quality control reagent in glycoprotein‐specific assays used to detect/identify alloantibody specificity. The authors describe the evaluation of an anti‐HPA‐15b lyophilized preparation coded 18/220 in an international collaborative study, the results of which are used to assign a minimum potency to this preparation. Clinical laboratories testing the established reference reagent at the assigned minimum potency should expect a positive result if the sensitivity of their method is acceptable.

## MATERIALS AND METHODS

### Candidate material

The material was provided to NIBSC by the National Blood Service, Oxford, United Kingdom. All donations of recalcified plasma came from just one consenting donor, and each was screened using the MAIPA to identify the anti‐HPA‐15b titre. Those with the highest titres were pooled to make a bulk (coded 18/220) prior to filling 0.5 ml/ampoule into 1896 glass ampoules (size 2.5 ml). Filled material was freeze‐dried under conditions established *in house* for the routine lyophilization of WHO serum standards/reference reagents; a summary of the product information is shown in Table 1. The individual donations from which the candidate reference material was prepared and the pooled bulk were found to be negative for HBsAg, anti‐HIV1 + 2 and anti‐hepatitis C virus (HCV). HCV PCR testing of the pooled bulk was also negative, and no microbial contaminants were detected.

**TABLE 1:** Product summary

Code number	18/220
Presentation	Heat sealed, 2.5‐ml glass ampoules
Number available	1830
Date filled	1 March 2019
Mean fill mass (*n* = 96)	0.52 g
Fill mass CV (*n* = 96)	0.65%
Residual moisture by coulometric Karl Fischer titration (*n* = 6)	0.20%
Residual moisture CV (*n* = 6)	24.8%
Mean dry weight (*n* = 6)	0.04 g
Dry weight CV (*n* = 6)	0.44%
Mean oxygen in head space by lighthouse FMS670 (*n* = 6)	0.20%
Oxygen in head space CV (*n* = 6)	44.69%
Storage conditions	−20°C
Address of processing facility and custodian	NIBSC, Potters Bar, UK

Abbreviation: CV, Coefficient of Variation.

A trial‐sized plasma pool containing the same proportions of plasma donations as for the definitive bulk (18/220) and selected single donations used to prepare 18/220 were evaluated by two independent clinical laboratories; both laboratories were able to detect anti‐HPA‐15b antibodies but could not detect antibodies with any other HPA specificity. Both laboratories also confirmed the presence of anti‐HLA class 1 antibodies, and the following specificities were detected: A2, A68, A69, C5, C8 and C15. Furthermore, constituent donations of the definitive pool have been evaluated in previous proficiency testing schemes organized by NIBSC; no other antibody specificities were reported other than anti‐HPA‐15b and anti‐HLA.

### Participants

The invitation was distributed to participants of the ISBT Platelet Immunology Workshop and to participants of the HPA antibody detection quality assessment scheme organized by the National External Quality Assessment Site UK (NEQAS). In total, 27 clinical laboratories located across the globe accepted the invitation to participate (see Table 2).

**TABLE 2:** List of participating laboratories

Institute	City, Country
Australian Red Cross Blood Service, Victoria	Melbourne, Australia
Australian Red Cross Blood Service, Queensland	Brisbane, Australia
Hospital Sirio Libanês	São Paulo, Brazil
Canadian Blood Services	Winnipeg, Canada
Institute of Blood Transfusion, Zhejiang Blood Centre	Hangzhou, China
Institute of Blood Transfusion, Guangzhou Blood Centre	Guangzhou, China
French Blood Establishment (EFS), HFNO	Lille, France
French Blood Establishment (EFS), Brittany	Rennes, France
French Blood Establishment (EFS), Aura	Lyon, France
Centre Hospitalier Universitaire de Nantes	Nantes, France
Institut National de la Transfusion Sanguine	Paris, France
Red Cross Blood Transfusion Services, NSTOB	Dessau, Germany
Zentrum für Transfusionsmedizin und Zelltherapie	Berlin, Germany
Institute of Clinical Immunology and Transfusion Medicine	Giessen, Germany
National Blood Centre	Kuala Lumpur, Malaysia
Sanquin Diagnostic Services	Amsterdam, Netherlands
University Hospital of North Norway	Tromsø, Norway
Institute of Haematology and Transfusion Medicine	Warsaw, Poland
Banc de Sang i Teixits	Barcelona, Spain
Karolinska University Hospital	Stockholm, Sweden
University Hospital Geneva	Geneva, Switzerland
The Thai Red Cross Society	Bangkok, Thailand
National Health Service Blood and Transplant	Filton, UK
Welsh Blood Service	Pontyclun, UK
National Institute for Biological Standards and Control	Potters Bar, UK
Versiti	Milwaukee, USA
Bloodworks	Seattle, USA

### Study design

Four ampoules of the definitive freeze‐dried material (coded 18/220) were sent to each laboratory. Participants were asked to reconstitute the material immediately before testing and to titrate at doubling dilutions in one or more assay method(s) routinely used in clinical practice. Participants were required to use a CD109 glycoprotein–specific method such as the MAIPA because the material contains anti‐HLA antibodies, which could otherwise cause false‐positive results. Clinical laboratories would normally be required to use such a method since patient samples can also contain HLA antibodies. Two ampoules were to be tested, each on a different day, with HPA‐15bb and HPA‐15aa platelets or similar on both days. Ideally, different platelet donors with the same HPA‐15 type were to be used over 2 days. Laboratories were asked to report their interpretation of the results for each test by recording ‘positive’ or ‘negative’ for each dilution tested. The endpoint dilution (antibody titre) for 18/220 in each test was assigned as the largest (maximum) dilution, which the laboratory reported to be ‘positive.’ Laboratories reporting results as ‘weak positive’ or results that were defined as borderline in ‘grey zones’ of defined optical density ranges were also deemed negative.

### Stability studies

To predict loss in stability over time, accelerated thermal degradation studies were performed at NIBSC using ampoules of lyophilized 18/220 stored at −70, −20, +4, +20, +37 and +45°C for 13 months. Reconstituted material from two ampoules at each temperature was tested in duplicate at a range of twofold serial dilutions in the MAIPA assay with HPA‐15bb platelets. Absorbance readings were used to calculate the relative potencies of the accelerated thermal degradation samples by parallel‐line analysis using the −70 sample as the reference. Relative potency calculations were performed using the European Directorate for the Quality of Medicines software CombiStats, version 6.0, using a sigmoid curve model and logit transformation of responses. The Arrhenius equation, relating degradation rate to absolute temperature assuming first‐order decay [16], was used to predict the degradation rates for each storage temperature.

## RESULTS

A total of 27 clinical laboratories accepted the invitation to participate in the study, however, only 25 laboratories returned results. Each laboratory was assigned a code number, which does not reflect the order of listing shown in Table 2. The methods used by participants are summarized in Table 3. All 25 laboratories performed glycoprotein‐specific assays, all of which were a version of the MAIPA using a monoclonal antibody specific for CD109. Some laboratories provided supplementary datasets from additional platelet donors or from a second method. A full summary of the results from each laboratory and details of their assay protocols are shown in Appendix S1.

**TABLE 3:** Laboratory method summary

Method	Laboratory code	Number of entries
Rapid MAIPA	3, 4^a^, 4a^a^ ^,^ ^b^, 5, 7, 7a^c^, 9, 11, 17, 17a^d^, 18, 21, 23	13
2‐Day MAIPA	1, 10^b^, 14, 16	4
In‐house MAIPA	2, 6, 12, 13, 15, 19, 20, 22	8
Other	8^b^ ^,^ ^e^, 18a^e^	2

*Note*: Labs providing supplementary datasets from a second method have an ‘a’ suffix.

Abbreviation: MAIPA, monoclonal antibody–specific immobilization of platelet antigen.

^a^ Frozen platelets.

^b^ CD109 antibody other than mAb TEA 2/16.

^c^ K562 Recombinant cells used.

^d^ Lyophilized platelets.

^e^ Modified MAIPA method.

### Reported endpoint dilutions against HPA‐15bb antigen

**FIGURE 1:**
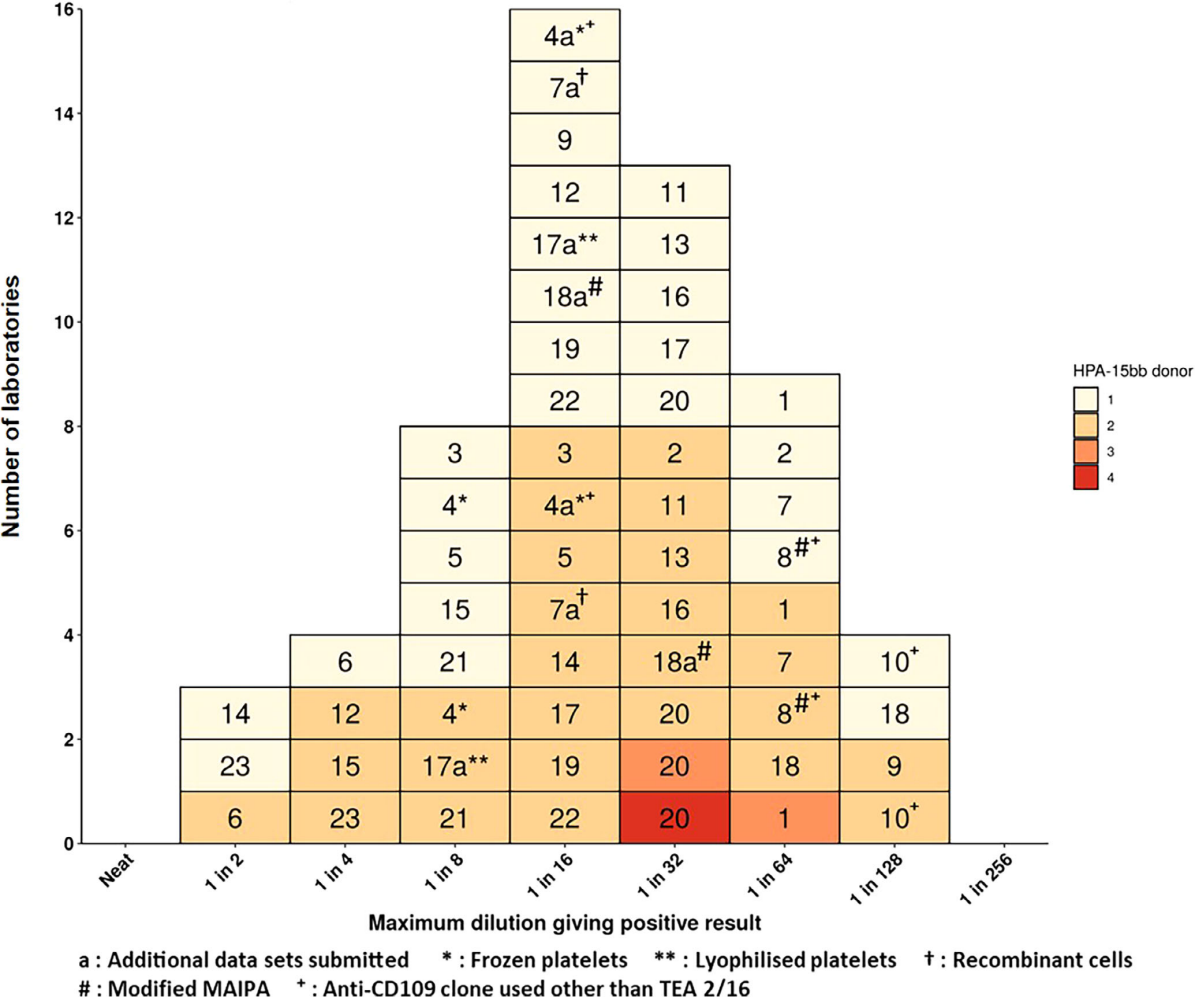
Maximum (endpoint) dilutions of anti‐HPA‐15b reference reagent (18/220) giving a positive result, as reported by international collaborative study participants for HPA‐15bb platelets/cells in CD109‐specific assays for the detection of anti‐HPA antibodies. Laboratory codes are shown in boxes; box colours refer to assay repetitions with different platelet donors except for 7 where the same donor for each repetition was used and 7a where recombinant cells were used

The majority of laboratories tested two ampoules of the material on two separate occasions, using different donors of HPA‐15bb platelets, as described in the study protocol. Laboratory 20 reported results from four different HPA‐15bb donors using three of the ampoules provided and laboratory 1 reported results using three different HPA‐15bb donors from three ampoules provided. Out of 25 laboratories, 24 were indeed able to detect antibodies with HPA‐15b specificity in preparation 18/220 with all HPA‐15bb platelet donors/cells used and in all methods performed. The remaining one laboratory reported a positive result for undiluted material using both HPA‐15aa and HPA‐15bb platelets but failed to titrate the material, and so endpoint dilutions could not be recorded. Another laboratory which did detect antibodies with HPA‐15b specificity failed to titrate the material enough; consequently, an endpoint dilution could not be recorded for this laboratory either. Figure 1 shows the endpoint dilutions reported by 23 laboratories for each method and for each donor of HPA‐15bb platelets/cells. The reported endpoint dilutions were normally distributed with a modal dilution of 1 in 16 and a range from 1 in 2 to 1 in 128 as shown in Figure 1. The intra‐laboratory variation in reported endpoint dilutions was significant in this study. Several laboratories (13 methods in total) reported different endpoint dilutions for 18/220 in each independent assay using the same method but where different HPA‐15bb platelet donors were used. For example, three of the five laboratories that reported poor endpoint dilutions (i.e., 1 in 2 or 1 in 4), only did so for one of two donors, with endpoint dilutions for a second donor of either 1 in 8 or 1 in 16. This difference could be attributed to the levels of CD109 expression on the platelets [13, 14]. The remaining two of the five laboratories reported 1 in 2 or 1 in 4 for both donors used.

The majority of laboratories used CD109‐specific, mouse monoclonal antibody TEA 2/16 from BD Pharmingen. There was no clear trend in reported endpoint dilutions for 18/220 relating to the different CD109‐specific monoclonal antibodies used as shown in Figure 1. As expected, most laboratories (22/23) used fresh HPA‐15bb platelets. Laboratory 4 used only frozen platelets characterized as having high CD109 expression levels prior to freezing and achieved an endpoint dilution in a MAIPA of 1 in 8 for 18/220 using the anti‐CD109 clone TEA 2/16 and an endpoint dilution of 1 in 16 using the anti‐CD109 clone HU17. Laboratory 17 compared lyophilized and fresh platelets in the MAIPA, with comparable endpoint dilutions being reported for both types (1 in 8 and 1 in 16 for two donors of lyophilized platelets and 1 in 16 and 1 in 32 for two donors of fresh platelets). Laboratory 7a used recombinant K562 cells with a reported endpoint dilution of 1 in 16.

Participants were asked to provide technical information on their protocols; this is summarized in Appendix S1. Indeed, there was considerable variation among the MAIPA methods performed, the most notable differences (further to those described earlier) being the number of platelets and sample volume used. Platelet numbers ranged from 0.4 × 10^6^ to 300 × 10^6^ per well and sample volumes ranged from 20 to 120 μl per well.

### Reported endpoint dilutions against HPA‐15aa antigen

**FIGURE 2:**
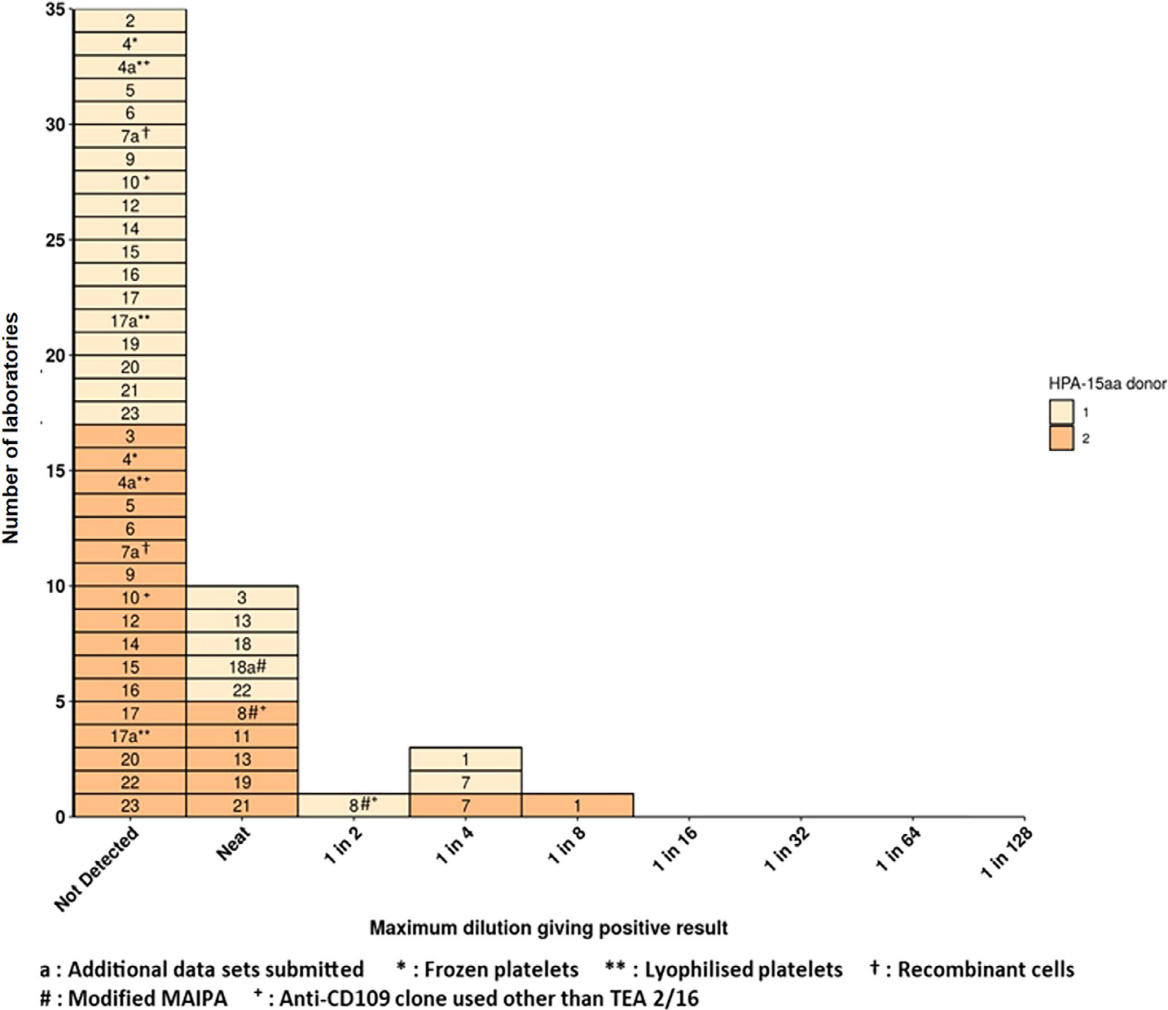
Maximum (endpoint) dilutions of anti‐HPA‐15b reference reagent (18/220) giving a positive result, as reported by international collaborative study participants for HPA‐15aa platelets/cells in CD109‐specific assays for the detection of anti‐HPA antibodies. Laboratory codes are shown in boxes; box colours refer to assay repetitions with different platelet donors except for 4, 4a and 7 where the same donor for each repetition was used and for 7a where recombinant cells were used

To confirm the specificity of the HPA‐15 antibodies within the candidate material, laboratories were also asked to test 18/220 with HPA‐15aa platelets from two different donors where possible at doubling dilutions and to report the endpoint dilution; these results are shown in Figure 2. There were only two laboratories that reported results from just one HPA‐15aa donor. In summary, as expected, nearly all laboratories reported that for all HPA‐15aa platelet donors (or alike) used with each method performed; they were unable to detect HPA‐15a antibodies or reported a positive result only for the undiluted (neat) material. However, three labs did report endpoint dilutions of 1 in 2, 1 in 4 or 1 in 8 with one or more donors. This could be explained by the presence of anti‐HLA antibodies at high serum concentrations and incomplete solubilization of the platelet membrane during the MAIPA procedure. Indeed, of both laboratories which reported endpoint dilutions of 1 in 4 or 1 in 8, each did so for different HPA‐15aa platelet donors suggesting that it is not a rare donor‐specific phenomenon but rather a procedural effect. One laboratory reported an endpoint dilution of 1 in 32 (result not shown in Figure 2); this appears to be anomalous in comparison with the results from all other laboratories. The anomaly could have arisen from the incorrect typing of the alleged HPA‐15aa platelets or may be attributed to the fact that these platelets were 9 days old. Indeed, the same laboratory reported ‘neat’ as the endpoint for a second donor of HPA‐15aa platelets which were only 6 days old. While some laboratories have reported a positive result with HPA‐15aa platelets at high concentrations of 18/220, there still remains a clear distinction between reported endpoint dilutions with HPA‐15bb and HPA‐15aa types.

### Stability

Estimates of the potency of 18/220 stored at elevated temperatures for a period of 13 months relative to 18/220 stored at −70°C for 13 months are summarized in Table 4. Tests for non‐parallelism and non‐linearity in the parallel‐line analysis to estimate relative potencies were not statistically significant (*p* = 0.14 and 0.59, respectively). There was insufficient degradation at the elevated temperatures, even after 13 months of storage, to fit the Arrhenius model, with only a clear relative potency loss at +45°C. This indicates that 18/220 will be stable for long‐term storage at −20°C and sufficiently stable to allow for shipment of ampoules at ambient temperature.

**TABLE 4:** Accelerated degradation studies

Storage temperature (°C)	Relative potency (−70°C reference, *n* = 4)	95% lower confidence limit	95% upper confidence limit
−20	1.00	0.92	1.09
+4	1.28	1.18	1.40
+20	1.13	1.04	1.23
+37	0.98	0.90	1.07
+45	0.72	0.66	0.79

*Note*: Ampoules stored for 13 months at each temperature.

## DISCUSSION

The aim of the study was to prepare and evaluate a stable, reference reagent for anti‐HPA‐15b detection that clinical laboratories can use to assess and validate the sensitivity of their routine assays. The collaborative study has shown that candidate preparation 18/220 contains anti‐HPA‐15b antibody that could be detected at a dilution of 1 in 8 by 21 of 23 laboratories with at least one donor. Setting the minimum potency at 1 in 8 dilution removes ambiguity resulting from positivity with HPA‐15aa platelets or alike at lower dilutions. The results of the study in general show good consistency across most laboratories but also indicate that some laboratories should consider further improving the sensitivity of their assay method. Since the material contains an anti‐HLA component (as may any patient clinical sample), it should only be used in techniques that are glycoprotein‐specific (i.e., for CD109) such as the ‘gold standard’ MAIPA which all laboratories used in this study or where it can be ensured that the anti‐HLA antibodies will not cause a false‐positive reaction (i.e., through chloroquine treatment of platelets to remove HLA‐class 1 epitopes).

A report of the 19th ISBT Platelet Immunology Workshop gave a comprehensive summary of the variations in the MAIPA procedure used by workshop participants and concluded that the MAIPA is far from harmonized which may contribute to variations in results [15]. While our study also showed a lack of inter‐laboratory harmonization of the CD109 MAIPA procedure, we were also able to demonstrate notable intra‐assay variability in reported endpoint dilutions that are likely due to the differential levels in CD109 expression by platelets from different donors, as previously reported [14]. Therefore, the harmonization of test methods using optimized assay conditions may well improve the sensitivity of testing across clinical laboratories to some degree but cannot overcome test variability as a result of donor–donor differences. The use of the anti‐HPA‐15b reference reagent will at least provide some guarantee as to the sensitivity of the platelets used.

All participants of the international collaborative study were invited to comment on the final study report, and their approval for use of 18/220 as a reference reagent for human IgG antibodies against HPA‐15b with a minimum potency of 1 in 8 was sought. No objections were received from the participants, and, in addition, this proposal was endorsed by the ISBT Working Party on Platelet Immunology. In October 2020, all data were reviewed by the WHO Expert Committee on Biological Standardization, and 18/220 was approved for use as a first WHO anti‐HPA‐15b reference reagent. This material should be used at a dilution of 1 in 8 for assay validation (i.e., the minimum potency which should test positive) and may be used to qualify ‘in‐house’ controls. It is hoped that wide use of this reference reagent will improve the sensitivity of test methods giving more confidence to the results generated.

## CONFLICT OF INTEREST

The authors declare no conflicts of interest.

## Supporting information


**Appendix S1.** Summary of results and assay information.
