# Supplementary material for: A first WHO reference reagent for the detection of anti‐human platelet antigen‐15b
Source: Vox Sang. 2021 Jun 23;117(2):275–81. doi: 10.1111/vox.13167 (PMC9292896; doi:10.1111/vox.13167)
Supplement: Supplementary file 1 — Appendix S1. Summary of results and assay information. [file VOX-117-275-s001.docx]

**Appendix S1.** Summary of results and assay information

| **Lab code** | **Method** | **Endpoint dilution HPA-15bb** | **Endpoint dilution HPA-15aa** | **Anti-CD109 mAb clone & supplier** | **Fresh, frozen or lyophilis-ed platelets** | **Platelet/**  **cell conc.**  **(x10^6^ per well)** | **Sample volume (µl/well)** | **Pos/neg cut-off criteria (as reported)** | **Comments** |
| --- | --- | --- | --- | --- | --- | --- | --- | --- | --- |
| 1 | 2-Day MAIPA | Donor 1:  1 in 64 | Donor 1:  1 in 4 | TEA 2/16 BD Pharmingen | Fresh | 200 | 50 | OD >0.2 | - |
|  |  | Donor 2:  1 in 64 | Donor 2:  1 in 8 |  |  |  |  |  |  |
|  |  | Donor 3:  1 in 64 | Donor 3: N/A |  |  |  |  |  |  |
| 2 | In-house MAIPA | Donor 1:  1 in 64 | Donor 1: not detected | TEA 2/16 BD Pharmingen | Fresh | 25 | 50 | OD >0.3 | Platelets used were either day 0 or day 1 |
|  |  | Donor 2:  1 in 32 | Donor 2: N/A |  |  |  |  |  |  |
| 3 | Rapid MAIPA | Donor 1:  1 in 8 | Donor 1: neat | TEA 2/16 BD Pharmingen | Fresh | 10 | 25 | OD >0.15; ratio >3 | - |
|  |  | Donor 2:  1 in 16 | Donor 2: not detected |  |  |  |  |  |  |
| 4 | Rapid MAIPA | Donor 1:  1 in 8 | Donor 1: not detected | TEA 2/16 BD Pharmingen | Frozen | 10 | 25 | OD >0.15; ratio >3 | HPA-15aa donors the same for day 1 & 2, panel of donors with high CD109 expression  identified for HPA-15 MAIPA. |
|  |  | Donor 2:  1 in 8 | Donor 2: not detected |  |  |  |  |  |  |
| 4a | Rapid MAIPA | Donor 1:  1 in 16 | Donor 1: not detected | HU17 Invitrogen | Frozen | 10 | 25 | OD >0.15; ratio >3 | HPA-15aa donors the same for day 1 & 2, panel of donors with high CD109 expression  identified for HPA-15 MAIPA. |
|  |  | Donor 2:  1 in 16 | Donor 2: not detected |  |  |  |  |  |  |
| 5 | Rapid MAIPA | Donor 1:  1 in 8 | Donor 1: not detected | TEA 2/16 BD Pharmingen | Fresh | 300 | 50 | Ratio >2 (to neg control) | - |
|  |  | Donor 2:  1 in 16 | Donor 2: not detected |  |  |  |  |  |  |
| 6 | In-house MAIPA | Donor 1:  1 in 4 | Donor 1: not detected | TEA 2/16 BD Pharmingen | Fresh | 20 | 50 | OD >0.2 | - |
|  |  | Donor 2:  1 in 2 | Donor 2: not detected |  |  |  |  |  |  |
| 7 | Rapid MAIPA | Donor 1:  1 in 64 | Donor 1:  1 in 4 | TEA 2/16 BD Pharmingen | Fresh | 25 | 50 | OD >0.16 | HPA-15bb and HPA-15aa donors the same for day 1 & 2 |
|  |  | Donor 2:  1 in 64 | Donor 2:  1 in 4 |  |  |  |  |  |  |
| 7a | Rapid MAIPA | Donor 1:  1 in 32 | Donor 1: not detected | Bio-Rad MCA1227 | Recomb-inant K562 cells | 25 | 50 | OD >0.28 | - |
|  |  | Donor 2:  1 in 32 | Donor 2: not detected |  |  |  |  |  |  |
| 8 | Modified MAIPA | Donor 1:  1 in 64 | Donor 1:  1 in 2 | BAF4385 R&D Systems | Fresh | 75 | 100 | MFI 3x neg control | Flow cytometric method with MFI readings |
|  |  | Donor 2:  1 in 64 | Donor 2: neat |  |  |  |  |  |  |
| 9 | Rapid MAIPA | Donor 1:  1 in 16 | Donor 1: not detected | TEA 2/16 BD Pharmingen | Fresh | 40 | 50 | OD >0.2 | - |
|  |  | Donor 2:  1 in 128 | Donor 2: not detected |  |  |  |  |  |  |
| 10 | 2-Day MAIPA | Donor 1:  1 in 128 | Donor 1: not detected | 15E10 / Sanquin | Fresh | 60 | 120 | OD >0.3 | - |
|  |  | Donor 2:  1 in 128 | Donor 2: not detected |  |  |  |  |  |  |
| 11 | Rapid MAIPA | *Donor 1:  1 in 32 | *Donor 1:  1 in 32^#^ | TEA 2/16 BD Pharmingen | Fresh | 10 | 25 | OD >3x+/-SD neg control | #Anomalous result not included, *day 9 platelets used, **day 3 platelets used, ***day 6 platelets used |
|  |  | **Donor 2: 1 in 32 | ***Donor 2: neat |  |  |  |  |  |  |
| 12 | In-house MAIPA | Donor 1:  1 in 32 | Donor 1: not detected | TEA 2/16 | Fresh | 100 | 20 | OD >0.2 | - |
|  |  | Donor 2:  1 in 4 | Donor 2: not detected |  |  |  |  |  |  |
| 13 | In-house MAIPA | Donor 1:  1 in 32 | Donor 1: neat | TEA 2/16 BD Pharmingen | Fresh | 20 | 50 | OD ≥0.1 (or mean neg control + 2SD if this >0.1) | CD109 levels measured by flow cytometry to check for adequate expression and comparable levels |
|  |  | Donor 2:  1 in 32 | Donor 2: neat |  |  |  |  |  |  |
| 14 | 2-Day MAIPA | Donor 1:  1 in 16 | Donor 1: not detected | TEA 2/16 BD Pharmingen | Fresh | 20 | 50 | OD >neg control + 2 SD | - |
|  |  | Donor 2:  1 in 2 | Donor 2: not detected |  |  |  |  |  |  |
| 15 | In-house MAIPA | Donor 1:  1 in 8 | Donor 1: not detected | TEA 2/16 BD Pharmingen | Fresh | 25 | 50 | OD >0.2 | - |
|  |  | Donor 2:  1 in 4 | Donor 2: not detected |  |  |  |  |  |  |
| 16 | 2-Day MAIPA | Donor 1:  1 in 32 | Donor 1: not detected | TEA 2/16 BD Pharmingen | Fresh | 40 | 50 | Ratio >2x neg control and OD >0.15 | - |
|  |  | Donor 2:  1 in 32 | Donor 2: not detected |  |  |  |  |  |  |
| 17 | Rapid MAIPA | Donor 1:  1 in 32 | Donor 1: not detected | TEA 2/16 BD Pharmingen | Fresh | 25 | 50 | OD >0.2 | - |
|  |  | Donor 2:  1 in 16 | Donor 2: not detected |  |  |  |  |  |  |
| 17a | Rapid MAIPA | Donor 1:  1 in 16 | Donor 1: not detected | TEA 2/16 BD Pharmingen | Lyophilis-ed | 25 | 50 | OD >0.2 | - |
|  |  | Donor 2:  1 in 8 | Donor 2: not detected |  |  |  |  |  |  |
| 18 | Rapid MAIPA | Donor 1:  1 in 128 | Donor 1: neat | TEA 2/16 BD Pharmingen | Fresh | 20 | 25 | 0.02 OD above negative control | - |
|  |  | Donor 2:  1 in 64 | Donor 2: N/A |  |  |  |  |  |  |
| 18a | Modified MAIPA | Donor 1:  1 in 16 | Donor 1: neat | TEA 2/16 BD Pharmingen | Fresh | 20 | 25 | 0.02 OD above negative control | - |
|  |  | Donor 2:  1 in 32 | Donor 2: N/A |  |  |  |  |  |  |
| 19 | In-house MAIPA | Donor 1:  1 in 16 | Donor 1: not detected | TEA 2/16 BD Pharmingen | Fresh | 4 | 50 | Ratio >2 | - |
|  |  | Donor 2:  1 in 16 | Donor 2: neat |  |  |  |  |  |  |
| 20 | In-house MAIPA | Donor 1:  1 in 32 | Donor 1: not detected | TEA 2/16 BD Pharmingen | Fresh | 100 | 50 | OD >0.2 | - |
|  |  | Donor 2:  1 in 32 | Donor 2: not detected |  |  |  |  |  |  |
|  |  | Donor 3:  1 in 32 | Donor 3: N/A |  |  |  |  |  |  |
|  |  | Donor 4:  1 in 32 | Donor 4: N/A |  |  |  |  |  |  |
| 21 | Rapid MAIPA | Donor 1:  1 in 8 | Donor 1: not detected | TEA 2/16 BD Pharmingen | Fresh | 20 | 25 | OD >0.2 | Day 1 platelets used |
|  |  | Donor 2:  1 in 8 | Donor 2: neat |  |  |  |  |  |  |
| 22 | In-house MAIPA | Donor 1:  1 in 16 | Donor 1: not detected | TEA 2/16 BD Pharmingen | Fresh | 20 | 25 | OD >0.2 | - |
|  |  | Donor 2:  1 in 16 | Donor 2: neat |  |  |  |  |  |  |
| 23 | Rapid MAIPA | Donor 1:  1 in 4 | Donor 1: not detected | TEA 2/16 BD Pharmingen | Fresh | 20 | 25 | OD >0.150 | - |
|  |  | Donor 2:  1 in 2 | Donor 2: not detected |  |  |  |  |  |  |
